# Root causes of extended length of stay and unplanned readmissions after orthopedic surgery and hand surgery: a retrospective observational cohort study

**DOI:** 10.1186/s13037-020-00249-3

**Published:** 2020-06-26

**Authors:** Morag Tolvi, Hanna Tuominen-Salo, Mika Paavola, Kimmo Mattila, Leena-Maija Aaltonen, Lasse Lehtonen

**Affiliations:** 1 Department of Otorhinolaryngology – Head and Neck Surgery, University of Helsinki and Helsinki University Hospital, P.O. Box 263, 00029 HUS, Helsinki, Finland; 2 Department of Anesthesiology, Intensive Care and Pain Medicine, University of Helsinki and Helsinki University Hospital, Helsinki, Finland; 3 Department of Orthopedics and Traumatology, University of Helsinki and Helsinki University Hospital, Helsinki, Finland; 4 Group Administration, University of Helsinki and Helsinki University Hospital, Helsinki, Finland; 5 Diagnostic Center, Hospital District of Helsinki and Uusimaa and University of Helsinki, Helsinki, Finland

**Keywords:** Readmission, Overstay, Revisit, Contact, Day surgery

## Abstract

**Background:**

While previous studies have evaluated the effect of some patient characteristics (e.g. gender, American Society of Anesthesiologists (ASA) class and comorbidity) on outcome in orthopedic and hand day surgery, more detailed information on anesthesia related factors has previously been lacking. Our goal was to investigate the perioperative factors that affect overstay, readmission and contact after day surgery in order to find certain patient profiles more prone to problemed outcomes after day surgery.

**Methods:**

We examined orthopedic and hand day surgery at an orthopedic day surgery unit of Helsinki University Hospital. Patient data of all adult orthopedic and hand day surgery patients (*n* = 542) over a 3-month period (January 1 – March 31, 2015) operated on at the unit were collected retrospectively using the hospital’s surgery database. These data comprised anesthesia and patient records with a follow-up period of 30 days post-operation. Patients under the age of 16 and patients not eligible for day surgery were excluded. Patient records were searched for an outcome of overstay, readmission or contact with the emergency room or policlinic. Pearson chi-square test, Fischer’s exact test and multivariable logistic regression were used to analyze the effect of various perioperative factors on postoperative outcome.

**Results:**

Various patient and anesthesia related factors were examined for their significance in the outcomes of overstay, readmission or contact. Female gender (*p* = 0.043), total amount of fentanyl (*p* = 0.00), use of remifentanil (*p* = 0.036), other pain medication during procedure (*p* = 0.005) and administration of antiemetic medication (*p* = 0.048) emerged as statistically significant on outcome after day surgery.

**Conclusions:**

Overstay and readmission in orthopedic and hand day surgery were clearly connected with female patients undergoing general anesthesia and needing larger amounts of intraoperative opioids. By favoring local and regional anesthesia, side effects of general anesthesia, as well as recovery time, will decrease.

## Introduction

Day surgery is an ever-growing field. As of 2016, 67% of surgical procedures in the United States were outpatient [1]. A procedure is considered suitable as day surgery when readmissions remain below 2–4% [2, 3]. A US study showed a 30-day readmission rate of 1.2% of orthopedic outpatients [4]. Overstay rates in orthopedic day surgery have been shown to be 0.1–0.8% [3, 5]. Pain and bleeding have been the most common reasons for patients returning to the hospital after day surgery in several studies [5–8].

In 2018, day surgery procedures totaled 36,897 in the Helsinki and Uusimaa Hospital District [9]. Helsinki and Uusimaa Hospital District comprises 23 hospitals with a catchment population of over 1.6 million, making it the largest hospital district in Finland. Forty percent of all day surgery in Finland is orthopedic and hand surgery (OHS) [10]. When procedurally possible, day surgery is usually performed under local or regional anesthesia in Finland. While previous studies have examined the effect of some patient characteristics (e.g. gender, American Society of Anesthesiologists (ASA) class and comorbidity) on outcome, information on anesthesia related factors has previously been lacking. Our goal was to investigate the perioperative factors that affect overstay, readmission and contact after day surgery in order to find certain patient profiles more prone to problemed outcomes after day surgery.

## Methods

### Study design

We studied orthopedic and hand (OHS) day surgery at Helsinki University Hospital with a special focus on overstay, readmission and contact rates. We examined the effect of local anesthesia and other perioperative factors on these outcomes. Patient data of all OHS day surgery patients (*n* = 542) treated during a 3-month period, between January 1, 2015 and March 31, 2015, at one of the three orthopedic day surgery units of Helsinki University Hospital, namely Herttoniemi Hospital, were collected retrospectively using the hospital’s surgery database (GE Healthcare Centricity Opera OR Management Software). These data comprised pre-, intra- and post-operative data and patient records with a follow-up period of 30 days post-operation. We chose the most common procedures using the Nordic Medico-Statistical Committee (NOMESCO) procedural codes, [11] grouped similar procedures (Table 1) and then divided them into sub-groups based on type of procedure: shoulder and elbow surgery, hand surgery and lower limb surgery.

**Table 1:** Table of day surgery procedures

**Orthopedic and hand surgery procedure name**	**No. of day surgery patients**	**Overall day surgery percentage in unit**
Decompression of median nerve, ACC51	167	96.5
Discission of sheath of tendon of wrist or hand, NDM40	46	97.9
Decompression of ulnar nerve, ACC53	43	97.7
Palmar fasciotomy of hand, NDM10	42	100
Incomplete excision of soft tissue tumor of wrist or hand, NDR20^a^	36	92.3
Arthroplasty of first CMC joint, NDG60	34	89.5
Excision of synovial ganglion of wrist or hand, NDM20	25	100
Fusion of DIP joint, NDG76	23	95.8
Open operation for osteochondritis of joint of wrist, NDF25	22	95.7
Arthroscopic exploration of joint of wrist or hand, NDA30	18	81.8
Radical excision of soft tissue tumor of wrist or hand, NDR30^a^	17	89.5
Partial fusion of wrist, NDG20	16	94.1
Arthroscopic partial excision of meniscus of knee, NGD05	16	88.9
Removal of internal fixation device from wrist or hand, NDU20	14	93.3
Removal of internal fixation device from shoulder or upper arm, NBU20	12	85.7
Plastic repair of ligament or capsule of wrist with transplant, NDE40	11	100

^a^combined to form excision of soft tissue tumor of wrist or hand group

In this study, overstay occurred if the patient was not discharged the same day as their day surgery procedure. Readmission was defined as a patient returning to the hospital after discharge and requiring treatment on the ward. A phone call or an outpatient visit to the emergency room or outpatient clinic was considered contact. Phone contacts are mentioned as most of these involved the prescribing of an antibiotic or the renewal of pain medication.

Finnish national law does not require ethics committee approval for registry studies with no patient intervention involved. Permission from the Research Administration of the Hospital District was obtained for this study.

### Participants

Participants were chosen for day surgery during the preoperative visit according to the day surgery criteria of our clinic (Table 2), which are in line with international standards [12]. Only these patients were included in this study. Orthopedic procedures on children under the age of 16 are performed at the Helsinki University Children’s Hospital, not at the orthopedic day surgery unit, and have therefore been excluded from this study.

**Table 2:** Day surgery criteria

Operation duration < 3 h	
No significant risk of respiratory tract swelling	
No respiratory tract anomalies	
No or stable chronic disease	
No obstructive sleep apnea	
Body mass index < 35	
Ability to climb more than 2 flights of stairs without stopping	
Ability to care for oneself independently	
No unstable psychiatric illnesses	
No drug or alcohol addiction	
Caregiver over the age of 16 at home for first postoperative night	

Data on patient demographics, including age, gender and ASA class, were gathered (Table 3) and patient charts were scrutinized with attention to overstays, readmissions and contacts within 30 days of day surgery. Anesthesia charts were examined for information on premedication, intraoperative and postoperative medication, as well as pain rating, nausea and other symptoms.

**Table 3:** Patient demographics in 542 day surgery procedures

	**Hand surgery**	**Shoulder & elbow surgery**	**Lower limb surgery**
*Patients* (n)	471	55	16
*Male* %	37.4	58.2	56.3
*Age* median (range)	53.8 (16.2–92.5)	54.3 (23.2–72.2)	42.2 (21.1–63.3)
*ASA class* median (range)	2 (1–4)	2 (1–4)	1 (1–3)
*Anesthesia*			
Local (%)	42.7	10.9	
Regional (%)	49.7	65.5	93.7
General (%)	7.6	23.6	6.3

The protocol of the clinic was followed in relation to anesthesia, surgical procedure, as well as treatment and prophylaxis of pain, nausea and vomiting. The form of anesthesia was chosen according to the protocol of our clinic, which favors local/regional anesthesia whenever medically possible. Day surgery procedures of distal upper and lower extremity are mainly performed under regional anesthesia while shoulder surgery is, due to the nature of the procedure, performed under general anesthesia. The form of anesthesia was discussed with the patient, and in selected cases general anesthesia was also available upon request of the patient. Patients received perioperative antibiotic prophylaxis if any implants were used (i.e. left inside the patient) and/or if the patient had any primary disease that could increase the risk for postoperative infection. The primary perioperative antibiotic of choice in our clinic was cefuroxime 1.5 g, and, in the case of hypersensitivity, the secondary choice was clindamycin 600 mg. Both were administered intravenously.

### Statistical analysis

Pearson chi-square test, Fischer’s exact test and multivariable logistic regression were used to analyze the effect of ASA class, age, gender, type of procedure, form of anesthesia, underlying medical conditions and medications, use of laryngeal mask airway versus intubation, use of various anesthesia drugs and analgesics pre-, intra- and postoperatively, pain rating on the Numeric Rating Scale (NRS) in the recovery room, body mass index (BMI), smoking status and whether the patient was hypotensive (systolic blood pressure < 100 mmHg) or hypertensive (systolic blood pressure > 140 mmHg) during the operation on the risk of any study outcome. Overstay, readmission and contacts were chosen as study outcomes. Factors significantly associated with the risk of overstay, readmission or contact were included in the multivariable model. *P*-values and adjusted odds ratios (OR) with 95% confidence intervals (CI) were used to express results. With the factors found significant or near significant, combination analyses were carried out to find risk profiles. For various combinations of risk factors, OR, sensitivity, specificity, positive predictive value (PPV) and negative predictive value (NPV) were evaluated. *P*-values less than 0.05 were judged to be statistically significant. The data were analyzed using IBM SPSS Statistics 25.0 (IBM. Corp., Armonk, NY).

## Results

During the 3-month period, 542 orthopedic and hand surgery patients underwent day surgery. The majority of selected procedures were performed as day surgery in our clinic (Table 1). Post-operative pain and nausea or vomiting were the causes for overstay overall. The causes for readmission were operation site abscess (*n* = 2 readmissions) and one patient each for epigastric pain and gastroscopy, intravenous line related infection, chest pain beginning after surgery and non-ST segment elevation myocardial infarction. All readmissions occurred in the upper limb surgery groups. The most common causes for contacts were operation site infection (*n* = 11 contacts), pain (*n* = 6), swelling (*n* = 5), problems with wound (*n* = 5), problems with cast (*n* = 4) and operation site bleeding (*n* = 4).

Ten percent (*n* = 5) of patients had an overstay or readmission after general anesthesia compared to 0.6% (*n* = 3) after local or regional anesthesia. Only two procedures did not involve any contacts, overstays or readmissions: radical excision of soft tissue tumor of wrist or hand (*n* = 17) and arthroscopic partial excision of meniscus of knee (*n* = 16). The overall readmission rate was 1.1%, overstay rate 0.7% and contact rate 9.0%. There were no deaths during the follow-up period.

Gender, fentanyl, other pain medication during procedure, remifentanil and antiemetic medication rose to statistical significance (*p* < 0.05) (Tables 4 and 5).

**Table 4:** Patient related factors and their effect on postoperative outcome in OHS day surgery

**Factor**	**No. Of patients**	**n (%) of outcomes**	***P*-value**
*Gender*	542		0.043
Male	217	9 (4.1)	
Female	325	28 (8.6)	
*Age (years)*	542		0.465
16–44	153	9 (5.9)	
45–64	292	20 (6.8)	
65–74	68	4 (5.9)	
75+	29	4 (13.8)	
*BMI (kg/m2)*	542		0.422
< 20	29	1(3.4)	
20–24.9	183	8 (4.4)	
25–29.9	176	16 (9.1)	
30.0–34.9	102	9 (8.8)	
35–39.9	32	1 (3.1)	
40–44.9	16	2 (12.5)	
45–49.9	3	0 (0)	
50–54.9	1	0 (0)	
*ASA class*	539		0.226
1	176	9 (5.1)	
2	228	13 (5.7)	
3–4	135	13 (9.6)	
*Cardiovascular disease*	542		0.96
yes	203	14 (6.9)	
no	339	23 (6.8)	
*Anticoagulant*	541		0.683
no	525	36 (6.9)	
warfarin	8	1 (12.5)	
aspirin	6	0 (0)	
NOAC	2	0 (0)	
*Diabetes*	542		0.17
yes	61	7 (11.5)	
no	481	30 (6.2)	
*Pulmonary disease*	542		0.413
yes	60	2 (3.3)	
no	482	35 (7.3)	
*Sleep apnea*	542		0.386
yes	20	0 (0)	
no	522	37 (7.1)	
*Migraine/headache*	542		0.576
yes	12	1 (8.3)	
no	530	36 (6.8)	
*Psychiatric condition*	542		0.512
yes	41	4 (9.8)	
no	501	33 (6.6)	
*Meniere/vertigo*	542		1
yes	3	0 (0)	
no	539	37 (6.9)	
*Immunosuppression*	542		0.639
yes	20	2 (10.0)	
no	522	35 (6.7)	
*Other underlying medical condition*	542		0.837
yes	118	7 (5.9)	
no	424	30 (7.1)	
*Smoking status*	534		0.549
no	378	22 (5.8)	
yes	140	12 (8.6)	
quit	12	1 (8.3)	
sometimes	4	0 (0)	

*BMI* Body mass index, *ASA* American Society of Anesthesiologists, *NOAC* Novel oral anticoagulant

**Table 5:** Anesthesia and analgesia drug related factors and their effect on outcomes in OHS day surgery

**Factor**	**No. Of patients**	**N (%) of outcomes**	***P*-value**
*Premedication paracetamol*	539		1
yes	510	35 (6.9)	
no	29	2 (6.9)	
*Premedication NSAID*	539		0.423
yes	339	21 (6.2)	
no	200	16 (8.0)	
*Premedication diazepam*	539		0.391
yes	226	18 (8.0)	
no	313	19 (6.1)	
*Oxycodone i.v. (mg)*	539		0.064
0–10	525	34 (6.5)	
> 10	14	3 (21.4)	
*Fentanyl i.v. (mg)*	539		0.00
0–0.15	502	29 (5.8)	
> 0.15	37	8 (21.6)	
*Other pain medication during procedure*	539		0.005
no	527	33 (6.3)	
NSAID (ketoprofen)	1	0 (0)	
paracetamol	3	2 (66.7)	
other strong analgesic (alfentanil, esketamine)	8	2 (25.0)	
*Remifentanil*	542		0.036
yes	48	7 (14.6)	
no	494	30 (6.1)	
*Propofol*	542		0.117
no	476	29 (6.1)	
general anesthesia	55	7 (12.7)	
sedation	11	1 (9.1)	
*Sevoflurane*	542		1
yes	1	0 (0)	
no	541	37 (6.8)	
*Glycopyrrolate*	542		0.656
yes	22	2 (9.1)	
no	520	35 (6.7)	
*Dexamethasone*	542		0.111
yes	45	6 (13.3)	
no	497	31 (6.2)	
*Rocuronium*	541		
yes	4	1 (25.0)	0.247
no	537	36 (6.7)	
*Antiemetic medication*	540		0.107
no	519	33 (6.4)	
ondansetron 4 mg	20	4 (20.0)	
metoclopramide 10 mg	1	0 (0)	
*Antiemetic medication*	540		
yes	21	4 (19.0)	0.048
no	519	33 (6.4)	
*Postoperative pain medication*	539		0.08
no	520	34 (6.5)	
NSAID (ibuprofen, ketoprofen)	8	0 (0)	
paracetamol	9	3 (33.3)	
weak opioids (paracetamol-codeine)	1	0 (0)	
strong analgesics (esketamine)	1	0 (0)	

*NSAID* Nonsteroidal anti-inflammatory drug, *i.v.* intravenous

Oxycodone, general anesthesia, plexus block and postoperative pain medication were borderline significant (*p* < 0.10) (Tables 5 and 6).

**Table 6:** Miscellaneous operation related factors and their effect on outcomes in OHS day surgery

**Factor**	**No. Of patients**	**n (%) of outcomes**	***P*-value**
*Procedure group*	542		0.835
Hand surgery	471	33 (7.0)	
Shoulder & elbow surgery	55	4 (7.3)	
Lower limb surgery	16	0 (0)	
*Laryngeal mask airway or intubation*	542		0.137
neither	488	30 (6.1)	
laryngeal mask airway	51	7 (13.7)	
intubation	3	0 (0)	
*General anesthesia*	542		0.077
yes	53	7 (13.2)	
no	489	30 (6.1)	
*Plexus block*	541		0.086
yes	166	16 (9.6)	
no	375	21 (5.6)	
*Intravenous regional anesthesia*	542		0.456
yes	114	6 (5.3)	
no	428	31 (7.2)	
*Spinal anesthesia*	542		0.617
yes	16	0 (0)	
no	526	37 (7.0)	
*Infiltrative anesthesia*	542		
yes	326	24 (7.4)	0.544
no	216	13 (6.0)	
*Peripheral nerve block*	542		1
yes	18	1 (5.6)	
no	524	36 (6.9)	
*NRS recovery room*	539		0.384
no pain 0	467	31 (6.6)	
mild 1–3	41	3 (7.3)	
moderate 4–6	27	2 (7.4)	
severe 7–10	4	1 (25.0)	
*Hypotensive during procedure*	540		0.794
yes	66	5 (7.6)	
no	474	32 (6.8)	
*Hypertensive during procedure*	540		0.959
yes	396	27 (6.8)	
no	144	10 (6.9)	

*NRS* Numerical rating system

These factors were joined in various combinations to find risk profiles for outcomes (Table 7). Other pain medication during procedure and postoperative pain medication were not included in the risk profile due to small sample size when combined with another risk factor.

**Table 7:** Various risk profiles for outcomes in OHS day surgery

**Combination**	**Total N**	**n of patients**	**OR (95% CI)**	***P*-value**	**PPV**	**NPV**	**Specificity**	**Sensitivity**
Female & Fentanyl ≥0.16 mg i.v.	539	16	6.97 (2.29–21.29)	< 0.001	31.3%	93.9%	97.8%	13.5%
Female & Remifentanil	542	18	4.25 (1.33–13.64)	0.015	22.2%	93.7%	97.2%	10.8%
Fentanyl ≥0.16 mg i.v. & Remifentanil	539	26	5.93 (2.31–15.21)	< 0.001	26.9%	94.2%	96.2%	18.9%
Female & Fentanyl ≥0.16 mg i.v. & Remifentanil	539	8	15.09 (3.61–63.06)	< 0.001	50.0%	93.8%	99.2%	10.8%
Fentanyl ≥0.16 mg i.v. & General anesthesia	539	30	4.86 (1.93–12.23)	< 0.001	23.3%	94.1%	95.4%	18.9%
Fentanyl ≥0.16 mg i.v. & Plexus block	538	18	5.87 (1.97–17.47)	0.001	27.8%	93.8%	97.4%	13.5%
Fentanyl ≥0.16 mg i.v. & Remifentanil & General anesthesia	539	26	5.93 (2.31–15.21)	< 0.001	26.9%	94.2%	96.2%	18.9%
Female & Fentanyl ≥0.16 mg i.v. & General anesthesia	539	10	10.02 (2.70–37.26)	< 0.001	40.0%	93.8%	98.8%	10.8%
Female & Fentanyl ≥0.16 mg i.v. & Plexus block	538	6	14.65 (2.85–75.33)	0.001	50.0%	93.6%	99.4%	8.1%
Female & Antiemetic medication	540	13	6.65 (1.95–22.75)	0.003	30.8%	93.7%	98.2%	10.8%
Fentanyl ≥0.16 & Antiemetic medication	539	14	2.33 (0.50–10.84)	0.280	14.3%	93.3%	97.6%	5.4%

*OR* Odds ratio, *CI* Confidence interval, *PPV* Positive predictive value, *NPV* Negative predictive value, *i.v.* intravenous

For each patient, we studied the form of anesthesia used (Table 1). The majority of orthopedic and hand surgery procedures were performed under regional or local anesthesia. All overstays and half of readmissions occurred in patients after general anesthesia. All of these readmissions and half of these overstays occurred in the group of 11 patients that underwent a partial fusion of the wrist with bone graft under general anesthesia. Eleven out of 49 contacts involved general anesthesia patients.

The standard premedication at the orthopedic and hand day surgery unit is paracetamol, etoricoxib and diazepam. Most patients received some combination of these three drugs. Premedication was adjusted according to allergies and possible medication in use. In addition to oxycodone, fentanyl and remifentanil, other pain medication administered during procedures included paracetamol, nonsteroidal anti-inflammatory drugs (NSAID) and other strong analgesics (Table 5). Postoperative pain medication included NSAIDs, paracetamol, weak opioids and strong analgesics. Pain was recorded in the recovery room on the NRS and the average of these answers for each patient was calculated (Table 6). The administration of antiemetic medication was significant (*p* = 0.048) for an outcome but the particular medication was not (*p* = 0.107).

## Discussion

Female gender, total amount of intraoperative and postoperative fentanyl, intraoperative administration of remifentanil, other pain medication during procedure and administration of antiemetic medication emerged as statistically significant. General anesthesia, plexus block, total amount of intraoperative and postoperative oxycodone and postoperative pain medication were borderline significant factors on outcome after day surgery.

### Risk profiles

Ten combinations of risk predictors for any outcome of overstay, readmission or contact produced significant odds ratios of over four to 15 times higher than patients with none of these risk predictors (Table 7). The highest risk was for two profiles: both profiles comprised females with a large amount of fentanyl; one administered with remifentanil, the other plexus block. Both of these profiles were capable of identifying half of patients with study outcomes. While the total amount of fentanyl cannot be predicted preoperatively, gender and the need for remifentanil and plexus block are known before surgery. Patients with these risk predictors need to be evaluated more thoroughly and postoperative contingency plans set into place. The suitability of these patients for day surgery must also be assessed.

### Overstay

Nausea and vomiting comprised the most common reasons for overstay. As many as 55% of day surgery patients experience post-operative nausea and vomiting [13]. Tracheal intubation and opioid use are both contributing factors to the onset of these symptoms, with female gender shown to triple the risk [14]. In our study, both overstays due to nausea and vomiting involved male patients. Nonetheless, both cases involved the factors found significant for outcomes: a large amount of oxycodone and fentanyl, use of remifentanil and general anesthesia. Our overstay rate was 0.7%, which is in line with a previous study in the United Kingdom showing an overstay rate of 0.79% after orthopedic day surgery [3].

### Readmissions and contacts

The most contacts and readmissions occurred in the hand surgery group with the most frequent reason being operation site infection, comprising a post-operative infection rate of 1.1%. Of these patients, two received perioperative antibiotic prophylaxis. Some patients had an underlying medical condition or factor, such as severe depression, diabetes or smoking, which could have predisposed to infection. Operation site infection in hand surgery is reported to vary from 0.36 to 3.8%, with one study reporting a rate as high as 10.7% [15–18]. Previous studies have shown US readmissions rates to be 1.2–2.5% for orthopedic day surgery [4, 6]. Our readmission rate of 1.1% is less than proposed in the guideline of the Royal College of Surgeons.

### Exceptions to day surgery criteria

While no obstructive sleep apnea and BMI < 35 are both day surgery eligibility criteria, 52 patients had a BMI greater than 35 and 20 had sleep apnea. Those with sleep apnea had no study outcomes, while of those with BMI > 35, only three patients had outcomes, which were related to a large amount of opioids, diabetes or trauma to operation site from falling. However, of all 52 obese patients, all except two were operated on under regional or local anesthesia and only one of these two had an outcome. Based on these data, it may be prudent to revisit the strictness of the BMI criteria.

### Gender

Female gender was a risk factor for outcomes. The majority (60%) of patients in this study were female. The most common procedure (30.8%) in this study was decompression of the median nerve, which is most typically performed on middle-aged women. In our study, women undergoing this procedure numbered 74.4%. Of procedures performed on women, 6.2% underwent general anesthesia, whereas of those performed on men, 13.8% underwent general anesthesia. Five of eight patients with overstays and readmissions were female. Most outcomes involving women were, however, for more minor problems than those of men. Women, in general, are more prone to use healthcare services more frequently [19–21]. This may explain why women had more outcomes in our study.

### Problematic procedures

Recuperation from two procedures, removal of internal fixation device from shoulder (NBU20) and partial fusion of wrist (NDG20), is significantly more painful than for other procedures included in this study and these patients require a greater amount of pain management at the day surgery unit and at home. The majority of these procedures were performed under general anesthesia due to their nature. Most of the overstays and readmissions in this study were from these two procedures. Of the six NDG20 patients with outcomes, five were operated on under general anesthesia and one with only a plexus block. During general anesthesia, remifentanil infusion and repeated doses of fentanyl are given typically during operations known to be painful, such as shoulder surgery or fusion of the wrist. Some may argue that these procedures are known to be painful and therefore not appropriate for day surgery. However, as long as this risk is acknowledged and the patient is informed that recuperation may be rocky, these procedures are possible to be carried out as day surgery.

### Limitations of the study

Our study has some limitations. The anesthesia charts of three patients were not found in their files but the majority of information necessary for the study was obtained from electronic patient charts. Only intraoperative information remained lacking. The majority of OHS patients belonged to the hand surgery group, thus skewing the sizes of the groups and interpretation of the data. This study was performed retrospectively. Therefore, we lack knowledge pertaining to possible visits to hospitals outside of our hospital district, to the patient’s own general practitioner or private healthcare producer. These visits are presumably infrequent as patients are directed to contact the hospital where the procedure was performed in case of post-operative issues. Primary care centers are also usually ill equipped to treat emergency issues, such as post-operative hemorrhage. Retrospective data may also lack some information due to charting errors or absent-mindedness. No patients were contacted in regard to their recuperation.

With general anesthesia as a borderline significant risk for outcomes, more and more procedures should be performed under local or regional anesthesia. However, this can only be achieved when medically and procedurally prudent. Due to the nature of shoulder surgery, general anesthesia with or without a plexus block is most often the anesthesia method of choice, despite the risk of increased outcomes. While females had more outcomes than males, this may be due to women using healthcare services more in general. Nevertheless, pre- and post-operative patient guidance is essential. Patients must be selected meticulously and their concerns, in regard to day surgery and form of anesthesia, listened to carefully.

## Conclusion

International day surgery selection criteria have been fine-tuned over the years. Our hospital follows these criteria and our low overstay and readmission rates validate them once again. Overstay and readmission were clearly connected with those OHS patients undergoing general anesthesia and needing larger amounts of intraoperative opioids. Nevertheless, due to the nature of some procedures and individual patient characteristics, general anesthesia cannot be completely phased out. Of course, it is impossible to prevent all post-operative complications and contacts but through meticulous patient instruction and selection, we can facilitate prompt emergent care. By favoring local and regional anesthesia, or when medically possible combining regional anesthesia with general anesthesia and thus decreasing the need of intraoperative opioids, side effects of general anesthesia, as well as recovery time, will decrease.

## Data Availability

The datasets used and analyzed during the current study are available from the corresponding author on reasonable request.

## Abbreviations

ASA: American Society of Anesthesiologists
BMI: Body mass index
CI: Confidence interval
i.v.: Intravenous
NOAC: Novel oral anticoagulant
NOMESCO: Nordic Medico-Statistical Committee
NPV: Negative predictive value
NRS: Numeric rating system
NSAID: Nonsteroidal anti-inflammatory drug
OHS: Orthopedic and hand surgery
OR: Odds ratio
PPV: Positive predictive value

## References

[1] American Hospital Association TrendWatch Chartbook 2018. 2018, https://www.aha.org/guidesreports/2018-05-23-trendwatch-chartbook-2018-chapter-3-utilization-and-volume.

[2] Royal College of Surgeons of England (1992). Commission on the Provision of Surgical Services.

[3] Johnson CD, Jarrett PE (1990). Admission to hospital after day case surgery. Ann R Coll Surg Engl.

[4] Jain U, Chandra RK, Smith SS (2014). Predictors of readmission after outpatient otolaryngologic surgery. Laryngoscope.

[5] Martin-Ferrero MA, Faour-Martin O, Simon-Perez C (2014). Ambulatory surgery in orthopedics: experience of over 10,000 patients. J Orthop Sci.

[6] Coley KC, Williams BA, DaPos SV (2002). Retrospective evaluation of unanticipated admissions and readmissions after same day surgery and associated costs. J Clin Anesth.

[7] Ansell GL, Montgomery JE (2004). Outcome of ASA III patients undergoing day case surgery. Br J Anaesth.

[8] Fortier J, Chung F, Su J (1998). Unanticipated admission after ambulatory surgery — a prospective study. Can J Anaesth.

[9] Helsingin ja Uudenmaan Sairaanhoitopiiri (HUS) (2018). Vuosikertomus 2018.

[10] Mattila K, Hynynen M, Group ICS (2009). Day surgery in Finland: a prospective cohort study of 14 day-surgery units. Acta Anaesthesiol Scand.

[11] NOMESCO (2016). Classification of surgical procedures.

[12] Association of Anaesthetists of Great Britain and Ireland; British Association of Day Surgery. Day case and short stay surgery: 2. Anaesthesia. 2011;66(5):417–34. 10.1111/j.1365-2044.2011.06651.x 21418041

[13] Wu CL, Berenholtz SM, Pronovost PJ (2002). Systematic review and analysis of postdischarge symptoms after outpatient surgery. Anesthesiology.

[14] Kenny GNC (1994). Risk factors for postoperative nausea and vomiting. Anaesthesia.

[15] Harness NG, Inacio MC, Pfeil FF (2010). Rate of infection after carpal tunnel release surgery and effect of antibiotic prophylaxis. J Hand Surg Am.

[16] Hashemi K, Blakeley CJ (2004). Wound infections in day-case hand surgery: a prospective study. Ann R Coll Surg Engl.

[17] Kleinert JM, Hoffmann J, Crain GM, et al. Postoperative infection in a double-occupancy operating room. A prospective study of two thousand four hundred and fifty-eight procedures on the extremities. J Bone Joint Surg Am. 1997;79:503–13. 10.2106/00004623-199704000-00005 9111394

[18] Platt AJ, Page RE (1995). Post-operative infection following hand surgery. Guidelines for antibiotic use. J Hand Surg Br.

[19] Thompson AE, Anisimowicz Y, Miedema B (2016). The influence of gender and other patient characteristics on health care-seeking behaviour: a QUALICOPC study. BMC Fam Pract.

[20] Nabalamba A, Millar WJ (2007). Going to the doctor. Stat Canada.

[21] Dunlop S, Coyte PC, McIsaac W (2000). Socio-economic status and the utilisation of physicians’ services: results from the Canadian National Population Health Survey. Soc Sci Med.

